# Endoscopic negative pressure therapy for duodenal leaks

**DOI:** 10.3389/fsurg.2023.1099457

**Published:** 2023-04-18

**Authors:** Dörte Wichmann, Dietmar Stüker, Ulrich Schweizer, Moritz Senne, Benedikt Duckworth-Mothes, Emanuel Zerabruck, Alfred Königsrainer, Jeannine Bachmann

**Affiliations:** ^1^ Department of General, Visceral and Transplantation Surgery at the University Hospital of Tübingen, Tübingen, Germany; ^2^ Interdisciplinary Endoscopic Unit at the University Hospital of Tübingen, Tübingen, Germany; ^3^ Working Group of Experimental Endoscopy, Development and Training of the Department of General, Visceral and Transplantation Surgery at the University Hospital of Tübingen, Tübingen, Germany; ^4^ Clinic and Outpatient Department for Surgery, University Hospital Right of the Isar, Munich, Germany

**Keywords:** endoscopic negative pressure therapy, postoperative insufficiency, endoscopic complication management, duodenal leaks, duodenal insufficiency

## Abstract

**Background and study aim:**

Endoscopic negative pressure therapy (ENPT) is well established in the treatment of perforations of various etiologies in the upper and lower gastrointestinal tract. For duodenal perforations exist only case reports and series. Different indications are possible for ENPT in duodenal position: primary therapy for leaks, preemptive therapy after surgery for example, after ulcer suturing or resection with anastomoses, or as second line therapy in cases of recurrent anastomotic insufficiencies with leakage of duodenal secretion.

**Methods:**

A retrospective 4-year case series of negative pressure therapy in duodenal position indicated by different etiologies and a comprehensive review of current literature on endoscopic negative pressure duodenal therapy are presented.

**Results:**

Patients with primary duodenal leaks *n*= 6 and with duodenal stump insufficiencies *n* = 4 were included. In seven patients ENPT was the first line and sole therapy. Primary surgery for duodenal leak was performed in *n* = 3 patients. Mean duration of ENPT was 11.0 days, mean hospital stay was 30.0 days. Re-operation after start of ENPT was necessary in two patients with duodenal stump insufficiencies. Surgery after termination of the ENPT was not necessary in any patient.

**Discussion:**

In our case series and in the literature, ENPT has been shown to be very successful in the therapy of duodenal leaks. A challenge in ENPT for duodenal leaks is the appropriate length of the probe to safely reach the leak and keep the open pore element at the end of the probe in place despite intestinal motility.

## Introduction

Research on endoscopic negative pressure therapy of the upper and lower gastrointestinal (GI) tracts began in 2000 when two Munich surgeons, R. Weidenhagen and U. Grützner, hypothesized that the effects of negative pressure wound therapy could be transferrable to the GI tract. The first successful experiment was conducted for rectal insufficiencies, wherein an open-pore polyurethane sponge with a perforated drain within it was placed inside the cavity of a rectal insufficiency. Negative pressure was built up using a closed vacuum drainage system (Redon) (1).

A device composed of an open-pore material, a perforated drain, and a connected negative pressure source is called an open-pore suction device (OPSD) (2). Endoscopic negative pressure therapy (ENPT) is also known as endoscopic vacuum therapy or endoscopic vacuum-assisted closure. The mode of functionality is always the same, i.e., an open-pore material surrounded the distal end of a perforated probe. The open-pore material including the probe is placed in an endoluminal or intracavitary position of an intestinal leakage. The end of the probe is drained through natural orifices (nose or anal sphincter) and is connected to a vacuum source (electronic pump or Redon system).

Currently, ENPT is widely used as endoscopic treatment for postoperative leaks and iatrogenic, spontaneous, and traumatic perforations of the upper and lower GI tracts. Different open-pore materials are used for probe preparation in ENPT. The classical material is the open-pore foam drainage (OPD), as described by Weidenhagen and Gruetzner, using a Polyurethane sponge (1). OPDs are commercial products available in Europe (Endo- and Eso-Sponge, B. Braun Melsungen AG, Melsungen, Germany). In 2015, Gunnar Loske introduced the CNP® film (Suprasorb CNP® Drainage Film, Lohmann & Rauscher International GmbH & Co.KG, Rengsdorf, Germany) (3). A cut-by-size CNP® film is wrapped around a perforated drain and fixed by sutures. This drainage type is called open-pore film drainage (OFD). Different tubes and drains with small diameters could be used for this technique. A case report described the creation of OPSD antimicrobial incised drape (4). Some techniques for the application the OPSD are proposed, and the push technique was used in most cases (5). In cases with transcutaneous fistula to the leaks, a pull-through-technique was used (6).

Duodenal ENPT, unlike esophageal or rectal applications, is not well described. It is possibly indicated for the treatment of iatrogenic duodenal perforations after endoscopic mucosal resection (EMR) or endoscopic retrograde cholangiopancreatography as preemptive therapy after suturing of duodenal ulcers and in cases of recurrent anastomotic insufficiencies after segmental duodenal resections. The OPSD is a handmade prototype in every case of duodenal ENPT placement. The major challenge associated with the use of ENPT for duodenal leaks is deciding the length of the probe to safely reach the leak and allow the open-pore element at the end of the probe to remain in place despite intestinal motility.

Herein, we present a case series of duodenal ENPT and describe the results of a comprehensive and current review of this topic.

## Material and methods

### Study design

All patients treated with ENPT in the duodenal position in the period between January 2018 and June 2022 were considered for inclusion in this study, given that the following criteria were met: confirmed diagnosis of duodenal leakages and its treatment at our department. The exclusion criteria were treatment without ENPT. This study focused on the management of postoperative complications; thus, patients who had undergone surgery in another hospital were included.

Informed consent and consent to participate were obtained from all patients. The local Institutional Review Board approved this study (IRB no. 751/2019BO2; Date of approval October 31, 2019).

### Etiology of the duodenal leaks

Patients with primary duodenal leaks as diverticula perforation (*n* = 2), patient with a duodenal perforation after EMR (*n* = 1), patients with duodenal leaks after suturing of perforated ulcerations (*n* = 3), and patients with duodenal stump insufficiencies after duodenopancreatic resections (*n* = 4) were included for this analysis. Patients with insufficiencies of the pancreaticogastric or gastrojejunal anastomosis were excluded.

### Endoscopic examination and application of the OPSD

Diagnostic and therapeutic endoscopies for leaks in the duodenal position were conducted in the suites of the interdisciplinary endoscopic unit or in the intensive care unit (ICU). Endotracheal intubation was not required in all cases. Endoscopes must be matched to the anatomical condition. In cases of duodenal stump insufficiency, children colonoscopes were used (OD 11.4 mm, length 160 cm). All OPSDs were handmade prototypes.

A small OPD was placed in one patient with spontaneously perforated duodenal diverticulum after 18 days with OFD. The perforated distal end of a nasobiliary probe was used to create the OPSD. The OPD was placed directly into the diverticulum. In a patient with duodenal perforation after EMR, a handmade OPD filling the lumen was placed.

**Figure 1:**
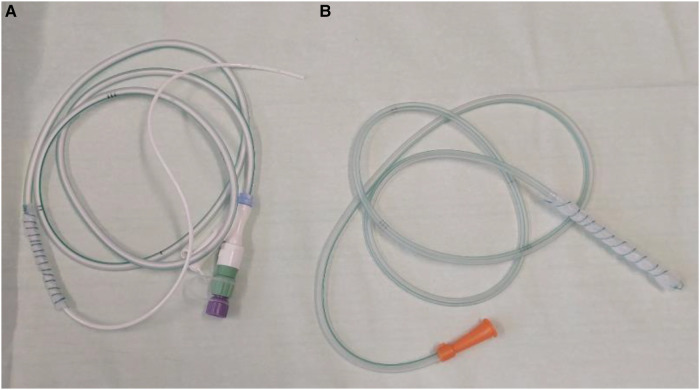
Two handmade prototype tubes for ENPT in duodenal/jejunal position. (**A**) For ENPT and enteralization: nasojejunal tube (Duodenal Tube Levin, 16 Ch, Dahlhausen, Cologne, Germany) with drainage film (Suprasorb CNP, Drainage Film; Lohmann & Rauscher International GmbH & Co. KG, Rengsdorf, Germany) wrapped on the distal segment. The white intestinal tube (9Ch, out from the Freka EasyIn® system Fresenius Kabi Deutschland GmbH, Bad Homburg, Germany). (**B**) For ENPT of insufficiencies of the biliary loop: nasojejunal tube (Duodenal Tube Levin, 16 Ch, Dahlhausen, Cologne, Germany) with drainage film (Suprasorb CNP, Drainage Film; Lohmann & Rauscher International GmbH & Co. KG, Rengsdorf, Germany) wrapped on the distal segment.

For the OFD, a very thin open-pore double-layered drainage film (Suprasorb CNP, Drainage Film; Lohmann & Rauscher International GmbH & Co. KG, Rengsdorf, Germany) was wrapped on the gastric segment of a nasojejunal feeding tube (Freka Trelumina, Fresenius Kabi Deutschland GmbH, Bad Homburg, Germany) or a modified nasojejunal tube with an intestinal tube (Duodenal Tube Levin, 16 Ch, Dahlhausen, Cologne, Germany) (Figure 1A) or for stump insufficiencies a nasojejunal tube (Duodenal Tube Levin, 16 Ch, Dahlhausen, Cologne, Germany) (Figure 1B) was used. Sutures (Mersilene, Polyester, four Ph. Eur., Ethicon, Norderstedt, Germany) were used to fix the drainage film around the tube. The OFD was placed using the push technique with a guide wire into the duodenum or jejunum. In some cases, additional endoscopic push maneuvers using endoscopic forceps were required. Endoscopic control of the correct placement of the tube was performed in every case.

The OFD was manufactured to cover the leak area with an overlap. The distal segment of the tube or the inserted intestinal tube was used for enteral feeding. Drains of the OPSD were oronasally redirected and fixed with plasters. After placement of the OFD, the drain was connected to an electric vacuum pump (KCI V.A.C. Freedom; KCI USA Inc., San Antonio, TX, USA), and a continuous vacuum of −125 mmHg was generated.

### Follow-up procedures

According to the clinical course and individual risk of the patients, follow-up examinations were mostly performed under sedation and only rarely under intubation anesthesia. Whenever possible, re-endoscopy was performed after 5–7 days in OFD-treated cases and 3–5 days in OPD-treated cases. In the case of persisting leak or uncertain cases, an OPSD was reinserted, and treatment was continued. In cases of leak closure the use of ENPT was determined. A diagnostic endoscopy was performed after three to five days after OPSD removal.

### Data analysis

Analysis was performed using IBM SPSS Statistics for Windows, version 24.0.0.1 (IBM Corp., Armonk, NY, USA). Data were presented as means ± standard deviation.

### Systematic review

**Figure 2:**
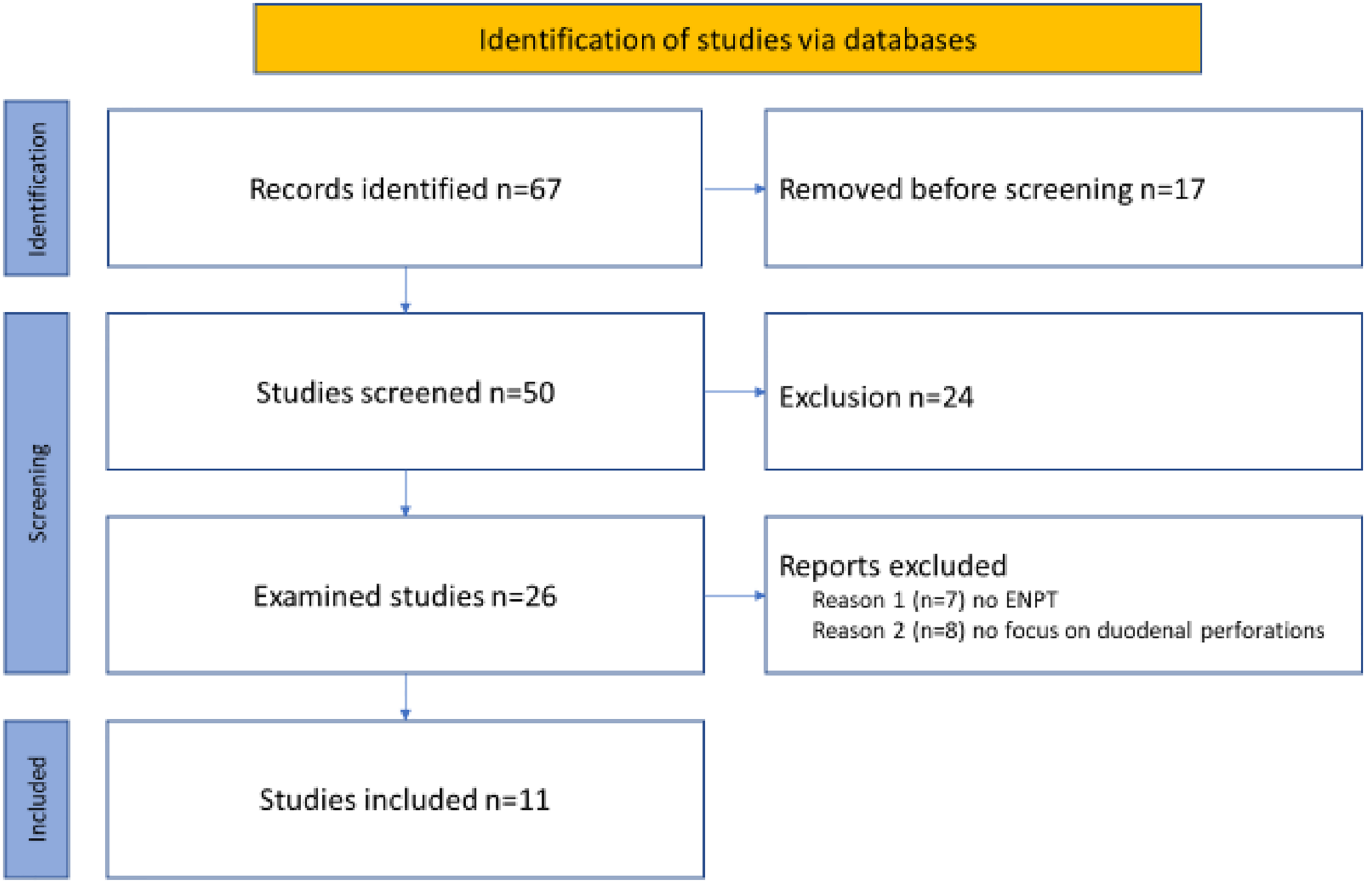
PRISMA 2020 flow diagram for the review process.

A literature search was performed according to the revised PRISMA guidelines of 2020 was performed by screening the electronic databases of MEDLINE (*via* Ovid SP), EMBASE (*via* DIMDI), Web of Science, and Cochrane Central Register of Controlled Trials (CENTRAL) covering the period from January 2010 to October 2022. A total of 43 studies were analyzed for this review. The flowchart of the review process is presented in Figure 2. Case reports of only one patient were not excluded from this review. The search strategies for the databases were adapted to the specific vocabulary of each database. Moreover, the references of the identified articles were manually screened to identify additional relevant studies. Two authors (DW and JB) independently reviewed the title and abstract of all records defined by the systematic literature search, and the full texts of all articles were assessed for eligibility.

## Results

### Retrospective analysis

A total of ten patients (6 males, 4 females; mean age 63.6 years) were included for the retrospective analysis. Table 1 presents the characteristics of the patients and etiologies of the perforations. Computed tomography of the abdomen was performed for all patients before primary endoscopic intervention. Primary endoscopic interventions were performed in seven cases at the ICU. All patients examined in the ICU received invasive ventilation. In two patients with spontaneously perforated duodenal diverticula, the primary endoscopy took place at the endoscopic suite without intubation narcosis. In all other patients, surgical interventions were performed for ulcerations or malignancies, not for the duodenal leakage. One patient was transferred from another hospital after surgery for perforated duodenal ulcers. All treated patients had covered perforations, i.e., retroperitoneal or ventral, but covered by adhesions. Duodenal insufficiencies of the biliary loop were treated in four patients after oncological duodeno-pancreatectomy. ENPT as primary therapy for duodenal/jejunal leaks was performed in seven of ten patients. Moreover, three of four patients with insufficiencies of the biliary loop were primary treated with surgical suturing. Two patients underwent surgery after the start of ENPT for duodenal leaks. Endoscopic therapy was initiated within 1 day after diagnosis.

**Table 1:** Patients’ characteristics.

Patient no.	Sex	Age	Year of diagnosis and therapy	Etiology of duodenal leak	Treated on ICU while diagnosis	Primary ENPT
1	F	69	2021	Perforated diverticula	No	Yes
2	F	82	2021	Perforated diverticula	No	Yes
3	M	48	2018	Leak after ulcer suturing	Yes	Yes
4	M	63	2020	Leak after ulcer suturing	Yes	Yes
5	F	75	2021	Leak after ulcer suturing	Yes	Yes
6	M	53	2019	Perforation after EMR	No	Yes
7	F	66	2018	Insufficiency of the biliary loop	Yes	No
8	M	63	2018	Insufficiency of the biliary loop	Yes	No
9	M	68	2019	Insufficiency of the biliary loop	Yes	Yes
10	M	49	2021	Insufficiency of the biliary loop	Yes	No
	**4:6**	**X̅ 63.6** **IQR 19.2**				

F, female; M, male; EMR, endoscopic mucosa resection; ICU, intensive care unit; ENPT, endoscopic negative pressure therapy, IQR, interquartile range.

Handmade prototype OPSDs were used based on nasojejunal feeding or nasojejunal tubes with a single lumen. OFD was used in all cases in endoluminally position. In one patient with perforated duodenal diverticula, an OPD was used on a nasobiliary probe after the placement of endoluminal OFD for 18 days. In a patient with duodenal leak after EMR, a handmade OPD was used for four days; thereafter, an OFD was placed endoluminally. In all cases, vacuum was set at 125 mmHg by an electric vacuum pump.

Enteralization was possible using a nasojejunal three-lumen feeding tube for the OFD. In patients with insufficiencies of the biliary loop, a nasojejunal single lumen probe was placed endoluminally into the loop. In these cases, enteral nutrition could not be provided and a total parenteral nutrition was necessary.

The duration of ENPT was dependent on laboratory parameters, progress radiology report about the amount of intra-abdominal fluids, clinical course, and endoscopic finding. A mean of 2.4 OPSD changes is necessary for a mean ENPT duration of 11.0 days. The mean hospital stays in the ten patients was 30.0 days, the duration of ICU stay was 8.0 days, and the patients received invasive ventilation for 3.1 days in mean. Table 2 presents that therapeutic course of the analyzed patients with duodenal perforation and ENPT.

**Table 2:** Therapeutic data of the included patients treated with ENPT for duodenal leaks.

Patient no.	Used OPSD	Duration of ENPT (d)/no. of changes	Enteralization while ENPT	Length of hospital stay/stay on ICU (d)	Duration of invasive ventilation (d)	Surgery after start of ENPT (*n*)	Clinical success
1	OFD	20/4	Yes	24/0	0	0	Yes
2	OFD + OPD	20/4	Yes, most of time	22/0	0	0	Yes
3	OFD	10/2	Yes	24/8	2	0	Yes
4	OFD	8/2	Yes	23/6	2	0	Yes
5	OFD	3/0	Yes	21/5	1	0	Yes
6	OPD + OFD	21/4	Yes	32/7	1	0	Yes
7	OFD	5/1	No	54/13	9	1 (lavage)	Yes
8	OFD	7/2	No	28/4	3	0	Yes
9	OFD	6/2	No	38/8	5	0	Yes
10	OFD	10/3	No	34/12	8	1	Yes
** **		**X̅ 11.0/2.4** **IQR 9.8/2**	**6**/**10**	**X̅ 30.0/8.0** **IQR 11.0/7.6**	**X̅ 3.1** **IQR 3.7**		

ENPT, endoscopic negative pressure therapy; OPSD, open-pore suction device; d, days; n, number, ICU, intensive care unit, IQR, interquartile range.

ENPT is terminated after endoscopic control and cross-sectional imaging in agreement with colleagues of the surgical and anesthesiology department. Re-operation after the end of ENPT was not needed in any patient.

### Systematic review

After excluding articles with duplicate use of patient data, a total of 11 articles on duodenal ENPT were identified (4, 7–16). The first description of ENPT in the duodenal position was published in 2010 (17). The largest case series by Loske et al. was published in 2019, which retrospectively analyzed 11 patients with duodenal leaks treated by ENPT (13). Nine articles were case reports or case series with 1–3 patients (4, 7–12, 14, 16).

Among OPSDs, OPD was used in most cases. De Moura et al. reported in a video article about the use of a cost-effective modified OPSD using an antimicrobial drape (4). ENPT was used as the first-line therapy for patients with duodenal leaks (*n* = 25; 75.76%). The overall success rate of ENPT in the duodenal position was 93.94%. Table 3 presents the analyzed parameters of the included case series. The clinical condition of the patients, which prohibited surgery, was mentioned in four articles (4, 12, 14, 16), and one case report described the lack of surgical options (7). The target negative pressure, which was generated by an electronic vacuum pump, was specified in all but one publication. It was 125 mmHg in seven (7, 10, 12–15), 175 mmHg in one (11), and 30 mmHg in two case articles (7, 9).

**Table 3:** Analyzed parameters of the included case series.

First authors name	Year of publication	Used OPSD	Patients number	Duration of ENPT	Number of OPSD changes	Primary ENPT	Success
Glatz et al. (7)	2015	OPD	1	20	3	1	1/1
Hochberger et al. (8)	2016	OPD	1	4	0	1	1/1
Yoo et al. (9)	2017	OPD	1	28	1	0	1/1
Kelm et al. (16)	2017	OPD	1	21	7	1	1/1
Mencio et al. (11)	2018	OPD	3	13.7	2.7	n.n.	3/3
Loske et al. (13)	2019	OFD + OPD	11	11	1,8	11	11/11
De Moura (4)	2021	Modified	1	n.n.	4	1	1/1
Wichmann et al. (10)	2021	OFD + OPD	2	16.5	4	2	2/2
Abbitt et al. (12)	2021	OPD	1	31	6	0	1/1
Martinho-Grueber et al. (14)	2022	OPD	1	21	7	1	1/1
Chevallay et al. (15)	2022	OPD	10	9	2	7	8/10
			**∑ 33**	**X̅ 17.52**	**X̅ 3.86**	**25/33**	**93.94%**

OPSD, open-pore suction device; OFD, open-pore film drainage; OPD, open-pore polyurethane-foam drainage; ENPT, endoscopic negative pressure therapy; n.n., not named.

Regarding the enteral status during ENPT for duodenal leaks, two articles reported enteralization *via* feeding tubes using OFD (10, 13), and a jejunostomy was used in one case report (16). Here, the OPD was placed retrogradely *via* the afferent loop, and a feeding tube was placed *via* the efferent loop (16). Two articles described the placement of the OPD through a percutaneous endoscopic gastrostoma (PEG) for better placement in the duodenal position (7, 14). These PEGs were not used for feeding. Four articles gave information about parenteral feeding during ENPT (8, 12, 14, 15).

The following aspects could not be consistently evaluated in the articles because of missing information: length of hospital stay, length of ICU stay, time from the diagnosis to the start of ENPT, OPSD-related complications, and follow-up results after discharge.

## Discussion

ENPT is an effective and easy-to-use endoscopic technique for the management of various GI leaks or insufficiencies (5, 18–20). Most previous studies have focused on ENPT for esophageal and rectal perforations and leaks. Since 2010, single case studies reported excellent outcomes of ENPT for duodenal leaks (17). The use of ENPT in the duodenal position was first described in patients who were in such a reduced clinical state that open surgery for the treatment of duodenal leakage was not possible (4, 7, 12, 14, 16). The most important effect of ENPT in the duodenal position is fluid removal as well as the removal of active digestive enzymes. Especially, the OFD is very suitable in fluid removal (2, 6).

ENPT is certainly not appropriate for all duodenal leaks: ENPT as first-line therapy is an option for leaks that drain to the retroperitoneum. Perforation that drains into the abdominal cavity must be surgically treated with lavage of the abdomen (21). In patients who underwent abdominal surgery and had periduodenal adhesions and to avoid drainage into the abdominal cavity, the ENPT could be an alternative to recurrent surgery. The results of our retrospective analysis and systematic review show that ENPT in the duodenal position can be extended over several weeks. During this time, patients may require further intensive care. For follow-up, CT data should be obtained in addition to endoscopic and clinical findings. Therefore, ENPT is always a resource-intensive treatment option. Prolonged perforations invariably result in leakage of duodenal secretions and a consecutive inflammatory reaction. In this case, not only a closure but also a drainage must be established. Chevallay and colleagues reported in their two-centric retrospective analysis about seven from ten patients which were treated with ENPT and additional CT-guided drains (15).

Alternative closure techniques for duodenal leaks are clip applications. Over-the-scope clip is an appropriate device to close leaks up to 2 cm (22). Multiple applications of hemoclips or the use of a hemoloop with clips can be effective endoscopic strategies to close duodenal perforations. These techniques are useful for endoscopically induced iatrogenic perforations that are directly discovered and treated. Hochberger et al. reported a large duodenal defect after endoscopic submucosal dissection (8). A clear perforation was not found but the denuded area was 4  ×  3 cm. The authors placed two OTSCs and some hemoclips, and to protect the mucosa from pancreatic fluids, an OPD was placed endoluminally. The follow-up control after 4 days showed adequate healing without suspected leak (8).

For endoscopists, the placement of the duodenal OPSD could be challenging. Different techniques can be employed when applying the OPSD in the duodenal or jejunal position. To reach the insufficiency by the endoscope could be challenging. We prefer the endoscopic examination in patients with a insufficiency of the biliary loop with a children colonoscope (OD 11.4 mm, length 160 cm). A limited aspect of duodenal/jejunal ENPT is the length of the probe and the safe position into the duodenum. For this reason, Mutagnini et al. described the successful use of non-coated nasojejunal probes in seven patients with duodenal stump insufficiencies and suction with a negative pressure between 80 and 100 mmHg (23). This article was not included to the review because OPSD was not used. The dislocation especially of the endoluminally placed OPSD is the most typical complication of duodenal ENPT. Two articles described the use of a PEG for OPD applications and changes. Another innovative technique would be possible: If a jejunostomy is created, the OPSD can be placed duodenally through it, and a feeding tube can be inserted into the draining limb. This procedure was successfully used and reported by Kelm et al. in 2017 in a patient with jejunal leakage after blunt injury (16).

All analyzed articles for the review were retrospective case reports or case series. This generates a substantial risk of publication BIAS. Furthermore, the present article joins the list as a retrospective case series. A prospective analysis would be advantageous, but will be difficult to implement due to the heterogeneous patient population and the highly patient-adapted approach.

The target negative pressure for ENPT is often 125 mmHg. In the review, two articles reported a negative pressure of 30 mmHg. However, these papers were published in 2015 and 2017, i.e., still in the early days of ENPT. Nevertheless, Jung et al. reported adequate healing of esophageal leaks using low negative pressure (20–50 mmHg) (18). However, the ideal negative pressure for wound healing by ENPT remains ambiguous.

The interval for OPSD changes is reported as 3–5 days. Thus, ENPT offers the possibility of close endoscopic control (6). In contrast, the majority of OPSD changes for duodenal perforations required intubation anesthesia. Hence, the condition of patients undergoing ENPT in the duodenal position is complex and they require interdisciplinary treatment with close consultation among surgery, radiology, gastroenterology, and anesthesia departments.

In summary, ENPT can be a good endoscopic tool for the treatment of duodenal/jejunal perforations as it can eliminate large quantities of fluids and active digestive enzymes. However, there are certain conditions to be met, i.e., the impossibility of open surgical treatment, retroperitoneal drainage of the leak, or abdominal adhesions that prevent the development of peritonitis. Endoscopically, ENPT is easy to use; in the duodenal position, the appropriate length of the OPSD probe is an important factor to be considered. Some innovative techniques for using PEG or jejunostomy have been described in literature.

## Data Availability

The original contributions presented in the study are included in the article/Supplementary Material, further inquiries can be directed to the corresponding author/s.
